# Alterins Produced by Oyster-Associated *Pseudoalteromonas* Are Antibacterial Cyclolipopeptides with LPS-Binding Activity

**DOI:** 10.3390/md18120630

**Published:** 2020-12-10

**Authors:** Florie Desriac, Abderrafek El Harras, Matthieu Simon, Arnaud Bondon, Benjamin Brillet, Patrick Le Chevalier, Martine Pugnière, Patrice Got, Delphine Destoumieux-Garzón, Yannick Fleury

**Affiliations:** 1 Laboratoire de Biotechnologie et Chimie Marine, EA3884, Université de Bretagne Occidentale, Université Bretagne Sud, 29334 Quimper, France; florie.desriac@unicaen.fr (F.D.); benjamin.brillet@univ-brest.fr (B.B.); patrick.lechevalier@univ-brest.fr (P.L.C.); 2 Institut des Sciences Chimiques de Rennes-CNRS-UMR 6226, Université Rennes, 35043 Rennes, France; abderrafek.el-harras@univ-rennes1.fr (A.E.H.); matthieusimon.work@gmail.com (M.S.); arnaud.bondon@univ-rennes1.fr (A.B.); 3 IRCM, Institut de Recherche en Cancérologie de Montpellier, INSERM U1194, Université de Montpellier, Institut régional du Cancer de Montpellier, 34298 Montpellier, France; martine.pugniere@inserm.fr; 4 MARBEC Université de Montpellier, CNRS, IRD, Place Eugène Bataillon CC 093, Place Eugène Bataillon, CEDEX 5, 34095 Montpellier, France; patrice.got@cnrs.fr; 5 Interactions Hôtes-Pathogènes-Environnements, Université de Montpellier, CNRS, Ifremer, Université Perpignan Via Domitia, 34095 Montpellier, France; delphine.destoumieux.garzon@ifremer.fr

**Keywords:** *Pseudoalteromonas*, cyclolipopeptides, alterin, antibiotic

## Abstract

Discovery after discovery, host-associated microbiota reveal a growing list of positive effects on host homeostasis by contributing to host nutrition, improving hosts’ immune systems and protecting hosts against pathogens. In that context, a collection of oyster associated bacteria producing antibacterial compounds have been established to evaluate their role in non-host-derived immunity. Here, we described alterins; potent anti-Gram negative compounds produced by *Pseudoalteromonas* h*Cg*-6 and h*Cg*-42 isolated from different healthy oyster hemolymph. The strains h*Cg*-6 and h*Cg*-42 produce a set of at least seven antibacterial compounds, ranging from 926 to 982 Da structurally characterized as cyclolipopeptides (CLPs). Alterins share the same cationic heptapeptidic cycle connected via an amido bond to different hydrophobic hydrocarbon tails. Their MICs disclosed a potent antibacterial activity directed against Gram-negative bacteria including oyster and human pathogens that may confer a beneficial defense mechanism to the host but also represents an untapped source of new antibiotics. The alterins’ mechanisms of action have been deciphered: after binding to lipopolysaccharides (LPS), alterins provoke a membrane depolarization and permeabilization leading to bacterial lysis. As h*Cg*-6 and h*Cg*-42 produced a set of natural derivatives, the structure/activity relationship linked to the carbon tail is clarified. We showed that the hydrocarbon tail determines the LPS-binding properties of alterins and consequently their antibacterial activities. Its length and saturation seem to play a major role in this interaction.

## 1. Introduction

Marine animals live in contact with a huge concentration of microorganisms [1] that they are forced to deal with through symbiotic, commensal or parasitic interactions. Benthic animals are particularly exposed due to their feeding-strategies (suspension-feeder, filter-feeder, deposit-feeder) and circulatory system (open versus closed). The past decade has seen the recognition of the critical role of microbial communities in host homeostasis. The hologenome theory has been brought into the spotlight [2,3]. Though controversial, this theory arises from host–microbial interactions in marine animals and *Cnidaria* in particular [2]. It assigned crucial functions to microbiota including genetic plasticity enhancement of the holobiont: a complex assemblage of the host and the associated-microbiota [4]. Host-microbial interactions are now well-documented for *Porifera* [5,6] and molecular dialogues have been investigated [7]. In bivalves, among which aquaculture species have been the most studied, knowledge and understanding of host-microbe interactions have mainly focused on pathogenesis. Nevertheless, recent data using high throughput pyrosequencing draw up the inventory of species composing healthy bivalve microbiome [8]. Tissue-specific microbiomes have been described in the oyster gut, stomach [9] and hemolymph [10,11]. However, the role of tissue-associated microbiomes remains unexplored in bivalves. 

Recently, we showed that about 3% of the culturable bacterial strains isolated from the hemolymph of healthy oysters, hereafter named *hCg*-strains, exhibit antibacterial activity against marine pathogens [12], suggesting that inter-bacterial competitions may contribute to oyster defense. The vast majority of them were affiliated to the *Pseudoalteromonas* genus [12,13]**, a well-documented genus for bioactive compounds production [14,15]. In addition, some *hCg*-strains displayed a dose-dependent beneficial effect on hemocyte survival rates suggesting that the hemolymph microbiota may play a role in bivalve immune homeostasis [13]. 

Here, we tested the microbial shield hypothesis by focusing on the hemolymph microbiome. We isolated and characterized, both structurally and functionally, the bioactive compounds responsible for the antibacterial activities of the *Pseudoalteromonas* strains associated with hemolymph. These compounds belong to a family of seven cyclolipopeptides (CLPs) composed of a macropeptidic cycle that harbours diverse hydrocarbon tails. The seven CLPs showed potent antimicrobial activity against Gram-negative bacteria including *Vibrio* strains pathogenic for a mollusc. We showed that they bind to bacterial lipopolysaccharides (LPS) and create damages to bacterial membranes. We propose to name them alterins. 

## 2. Results

### 2.1. Purification of Antibacterial Peptides

**Figure 1:**
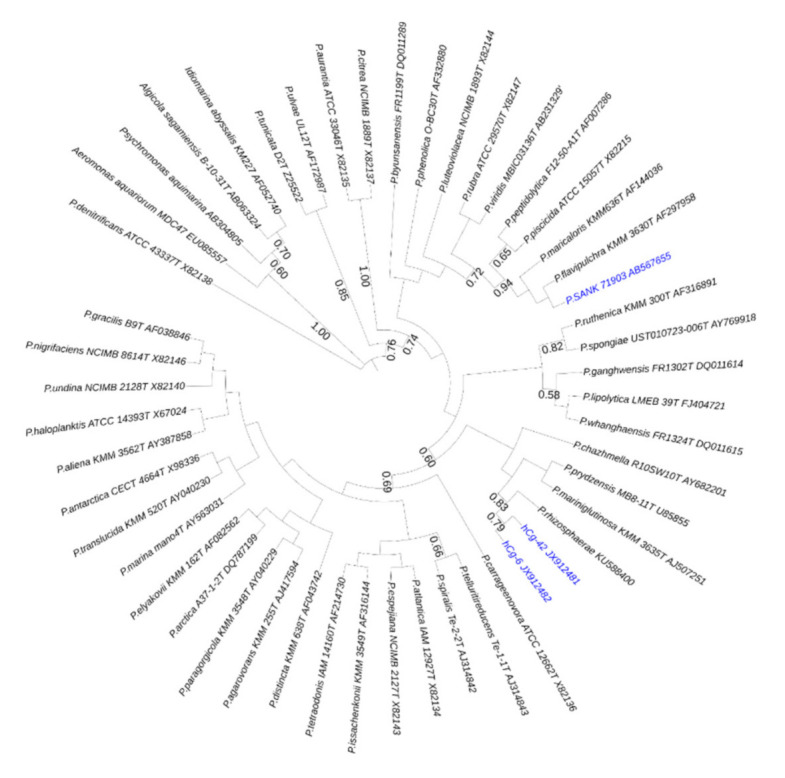
Maximum likelihood tree using the Tamura-Nei model [16,17] indicating the phylogenetic relationships inferred from partial 16S rRNA gene sequences of alterin-producing *Pseudoalteromonas* strains (in blue) within the *Pseudoalteromonas* genus. Bootstrap values (expressed as percentage of 1000 replications) >50% are shown at branching point. A group of four bacteria was used as outgroup and tree was constructed using MEGA X [18].

**Figure 2:**
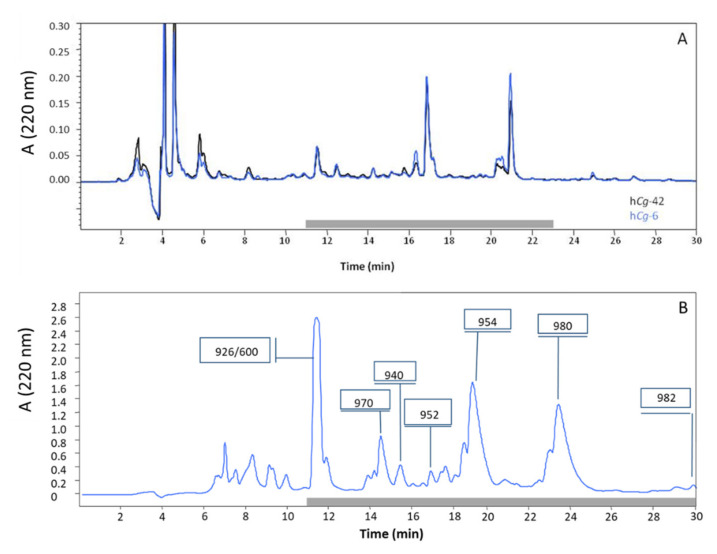
RP-HPLC analyses of the cell-free supernatants of *Pseudoalteromonas hCg*-6 (blue) and *hCg*-42 (black) strains. (**A**) Analytic chromatograms of cell-free supernatants (150 µL) of *Pseudoalteromonas hCg*-6 (blue) and *hCg*-42 (black). The grey bar indicates antibacterial activity. (**B**) Semi-preparative chromatogram arising from the injection of the bioactive fraction (2 mL) resulting the solid phase extraction of *Pseudoalteromonas hCg*-6 cell-free supernatant. The values framed indicate the m/z values resulting from MS analyses.

Hemolymph-associated microbiota of oysters (Crassostrea gigas) was explored for antibacterial activity against important aquaculture pathogens. We focused on two strains exhibiting the broadest and strongest antibacterial activities. Both of them belong to the genus Pseudoalteromonas. They were named hCg-6 and hCg-42 to reflect their origin hemolymph of Crassostrea gigas [12]. The two strains were shown to be phylogenetically closely related (Figure 1). To define the chemical structure of the bioactive compounds, a synthetic culture medium was developed to enhance antibacterial activity. After a 3-day long incubation at 18 °C, the cell-free supernatants were loaded on C_18_-SPE cartridges and analyzed using RP-HPLC. Both these hemolymph-associated strains displayed a similar chromatographic profile (Figure 2A). Purification was conducted using semi-preparative RP-HPLC (Figure 2B). Antibacterial fractions were further analyzed by mass spectrometry. The strains Pseudoalteromonas hCg-6 and hCg-42 produce at least seven active compounds whose molecular masses range from 926 to 982 Da. The Edman automated degradation analyses did not provide interpretable data to define an amino acid sequence. Consequently, the main produced bioactive compounds (eg compounds of 926, 940, 952, 954, 970, 980 and 982 Da) were subjected to NMR analyses.

### 2.2. Structural Characterization of Active Peptides

We defined the chemical structure of the antibacterial peptides by NMR analyses using standard sequential assignment procedure by combining 2D homonuclear (TOCSY and ROESY) and heteronuclear (^13^C, HQC and HMBC) spectra. A ROESY spectrum was used because of very weak signals with the NOESY sequence due to the intermediate molecular tumbling of the molecule as well as the absence of defined structure in water.

**Figure 3:**
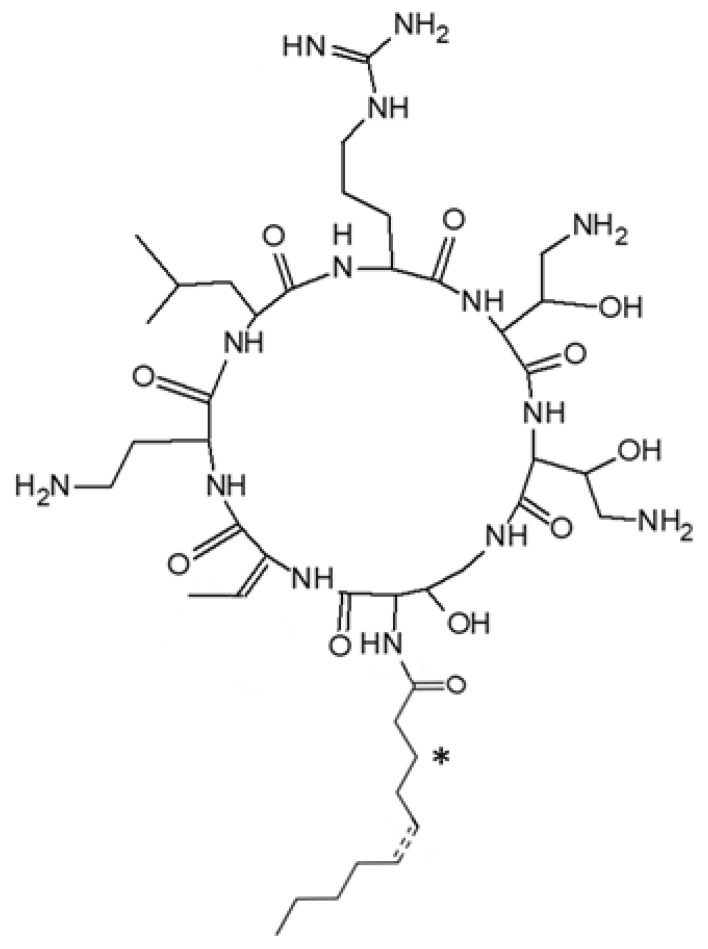
Chemical structure of the alterins. from *Pseudoalteromonas hCg*-6 and *hCg*-42 strains. *: hydroxylation at C_3_ position.

Pseudoalteromonas strains h*Cg*-6 and h*Cg*-42 were found to produce a series of cationic CLPs. All isolated CLPs share the same heptapeptidic moiety composed of two proteinogenic (Leu and Arg) and five exotic amino acid residues: a 2,4-diaminobutyric acid (Dab), a dehydrobutyrine (Dhb) and three 2 hydroxy-diaminobutyric acid (OH-Dab) (Figure 3). This monomer is quite rare even among the exotic residues of the non-ribosomal peptide (NRP), and was only described in odilorhabdin and ogipeptin [19,20].

However, the isolated CLPs differ from each other by their fatty acid moieties (Table 1). Mass changes arise from variation in fatty acid length (C8 to C12), unsaturation in 5′ position or hydroxylation in 3′ position (Figure 3). These structural characteristics are reminiscent of ogipeptins, a family of CLPs produced by Pseudoalteromonas sp. strain SANK 71903, which was recently characterized by Hirota-Takahata et al. [20]. However, the stereochemistry of the Dhb was different in CLPs produced by Pseudoalteromonas strains hCg-6 and hCg-42, which carry a E unsaturation in the Dhb side chain, and in ogipeptins, which carry a Z unsaturation. Therefore, despite this difference, we propose herein to rename all these CLPs, alterins. 

### 2.3. Antibacterial Activity

Antibacterial activity was assayed against a panel of micro-organisms including human and aquaculture pathogenic bacterial species. MICs were performed and defined according to internationally recognized standards (CLSI [21]). Polymyxin B (PMB), an antibiotic CLP produced by *Paenibacillus polymyxa*, was used as positive control. The major produced alterins, i.e., alt_952, alt_954, alt_970 and alt_980 were assayed against the whole panel of target cells while the minor ones were only tested against a few bacterial strains (Table 2). Since previous assays using the cell-free culture supernatants of the strains *hCg*-6 and *hCg*-42 did not reveal antibacterial activities against Gram positive bacteria and yeast [12], we focused on Gram negative bacteria and mainly marine pathogenic bacteria.

PMB and alterins exhibited a similar spectrum of activity being active against all the target bacteria except for the producing strains, *Pseudoalteromonas hCg*-6 and *hCg*-42 and the clam pathogen *Vibrio tapetis* CECT 4600 (Table 2). Moreover, alterins and PMB displayed overall the same MICs (µM range) except against *Vibrio harveyi* ORM4 that is more sensitive to alterins than to PMB. Even more markedly, *Vibrio parahaemolyticus* 13028A/3 is sensitive to alterin (with MIC of 6.25 µM) and resistant to PMB up to 100 µM (Table 2). Conversely, *Pseudomonas aeruginosa* ATCC27853 is susceptible to PMB (MIC 1.6 µM) and only partially sensitive to specific alterins (MIC of 22 µM to >100 µM).

MIC data shed light on structure-activity relationships. On average, alt_954, alt_980 and PMB were three to five times more potent than alt_952 and alt_970. This revealed that the insertion of an unsaturation (alt_952) or a hydroxylation (alt_970) into the hydrocarbon tail reduced the overall activity while a two carbon-long extension of the hydrocarbon tail (alt_980) appeared to restore the antibacterial potency when compared to alt_952. As noted above, this global trend has some exceptions depending on both the target strain and the specific alterin. For instance, *E. coli* SBS363 is more sensitive to alt_970 than alt_954. Interestingly, the *E. coli* SBS363 and ML35 strains which are known to expose short-chain and long-chain LPS respectively [22], present a discrepancy of sensitivity that may result from different bacterial outer-membrane structures. Therefore, we next investigated the direct interaction between LPS and two alterins, that is to say, alt_954 and alt_970.

### 2.4. LPS Binding Properties

**Figure 4:**
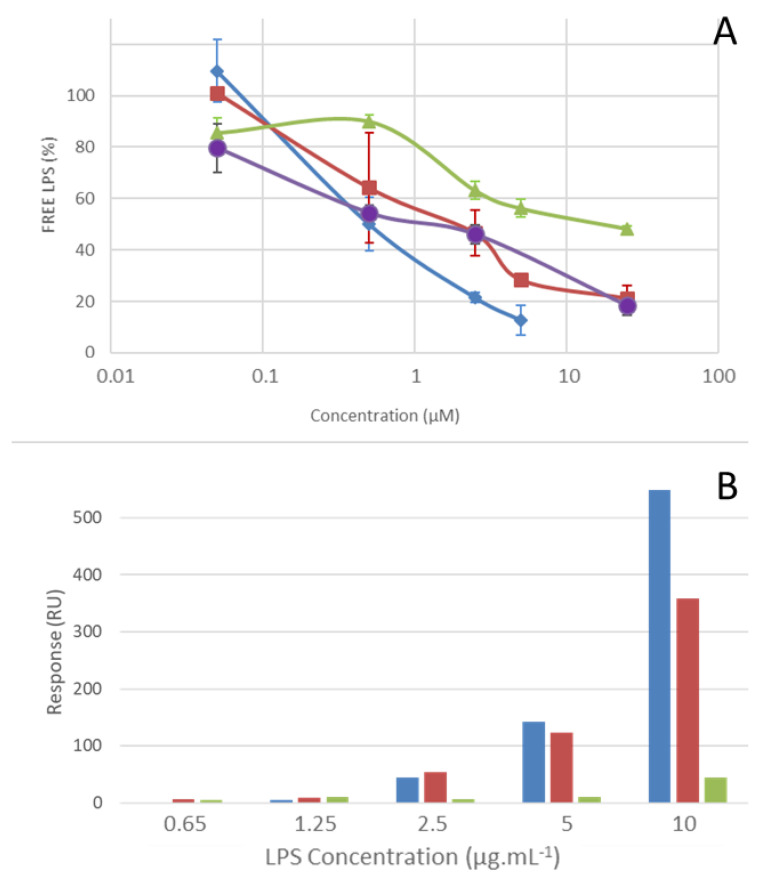
lipopolysaccharide (LPS)-binding properties of alterins. (**A**) Dose-dependent effects of alt_954 (■), alt_970 (▲), alt_980 (●) and Polymyxin B (PMB) (◆) on LPS detection using the *Limulus* Amoebocyte Lysate (LAL) assay. (**B**) LPS-binding of alt_954 (■), alt_970 (■) and PMB (■) expressed as resonant units (RU).

To gain insights into LPS-alterin interactions, we used an endotoxin detection assay, the QCL-1000^TM^ Endpoint chromogenic *Limulus* Amoebocyte Lysate (LAL) assay (Lonza). We compared alt_954, alt_970 and alt_980 for their ability to bind LPS from *E. coli* O111:B4. All alterins bound to LPS in a dose-dependent manner, as indicated by the reduction of biologically active LPS with increasing alterins concentrations (Figure 4A). As expected, alt_954 and alt_980 bound LPS similarly and more efficiently than alt_970. To examine alterin LPS-binding properties in more detail, we performed a surface plasmon resonance (SPR) assay using the Biacore technology (GE Healthcare). The SPR binding measurements were performed using the alt_954 and alt_970. PMB was used as a positive control. The CLPs were immobilized to a CM5 sensor chip surface and titrated with LPS. Alt_954 and PMB exhibited similar LPS-binding properties (Figure 4B) whereas in the same conditions, the response was halved with alt_970. The dose-dependent effect observed (Figure 4B) highlights the LPS-binding properties of the alterins. Both LPS-binding assays, i.e., LAL and SPR, are fully agreeing for alt_954. Moreover, the LPS-binding properties of alterins followed the same general pattern as that of antibacterial activity. This strongly suggests that alterin antibacterial activity depends on their LPS-binding ability.

### 2.5. Tridimensional Structure in LPS Micelles

**Figure 5:**
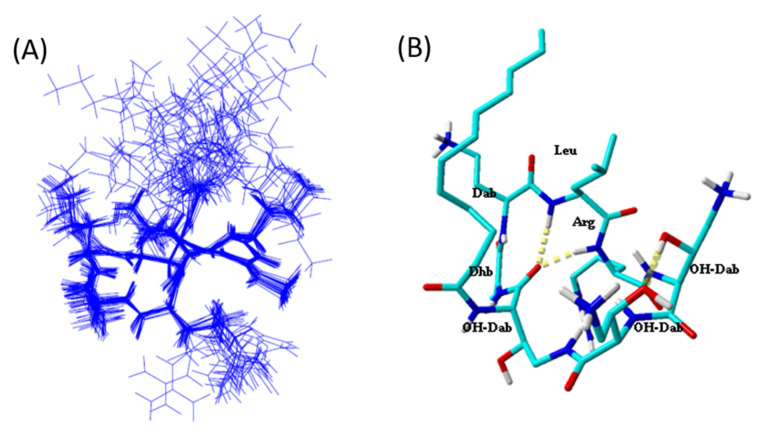
LPS-bound structure of alt_980. (**A**) Overlay of the 20 lowest energy structures with no violations >0.3 Å from the 100 structures calculated with AMBER software for alt_980 in presence of LPS micelles. Overlay was performed using the backbone atoms of the peptidic cycle. (**B**) Detail of the lowest energy structure displaying the hydrogen bonds between carbonyl of 7-OH-Dab and amide protons of 3-Leu and 4-Arg. Aliphatic protons are omitted for clarity.

The alterin-LPS interactions were then investigated through NMR. TrNOE is a widely used method that allows the 3D structure determination of small molecules interacting with large proteins or micelles [23,24]. We detected numerous negative transferred NOEs (trNOE) cross-peaks when alterins were in contact with LPS micelles, in contrast to what was observed in water solution. A total of 71 distance constraints from the NOEs of the Tr-NOESY spectrum were collected. For the 3D structure determination, we used all L-configuration accordingly with the data of Hirota-Takahata et al. [20]. Concerning the additional chiral α-positions of the three OH-Dab residues, both L and D configurations were used for molecular modeling. Structure convergence occurred only when the OH-Dab α-carbons were in an L configuration. All the dihedral angles are in the allowed region of the Ramachandran plot [25]. RMSD on the cyclic backbone atoms was 0.135 Å and 0.7 Å for all cyclic heavy atoms. The absence of NOESY cross-peaks involving the fatty acid side chain is responsible for its free orientation relative to the peptidic cycle. Figure 5 displays the overlay of the 20 lowest energy structures with no violations >0.3 Å from the 100 structures calculated with AMBER software for alt_980 in presence of LPS micelles. The overlay was performed using the backbone atoms of the cyclic heptapeptidic residues. Our data show that the cyclic structure is governed by a *β*-turn involving hydrogen bond between the carbonyl of 7-OH-Dab and amide protons of 3-Leu and 4-Arg. 

### 2.6. Membrane Effects and Bactericidal Activity of Alterins

**Figure 6:**
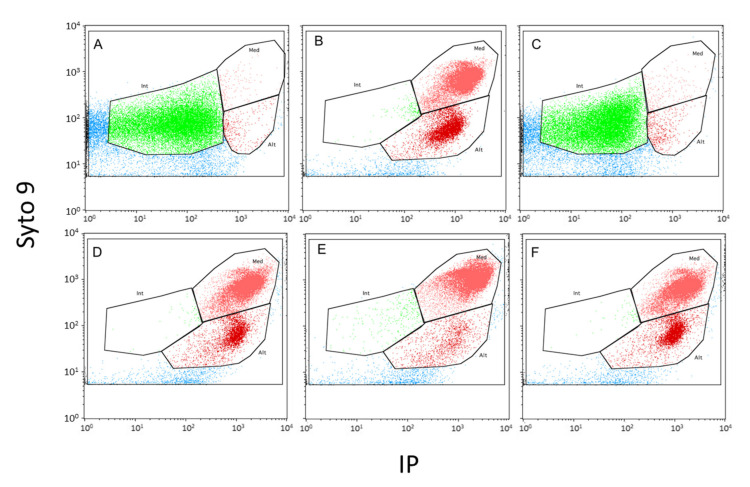
Flow cytometry analyses of *Vibrio tasmaniensis* LGP32 after a 4h-hour-long incubation with alterins (20 µM). (**A**) without antibacterial peptide in reconstituted seawater (negative control), (**B**) with PMB (20 µM) (positive control), (**C**) with alt_952, (**D**) with alt_954, (**E**): with alt_970 and (**F**) with alt_980.

**Figure 7:**
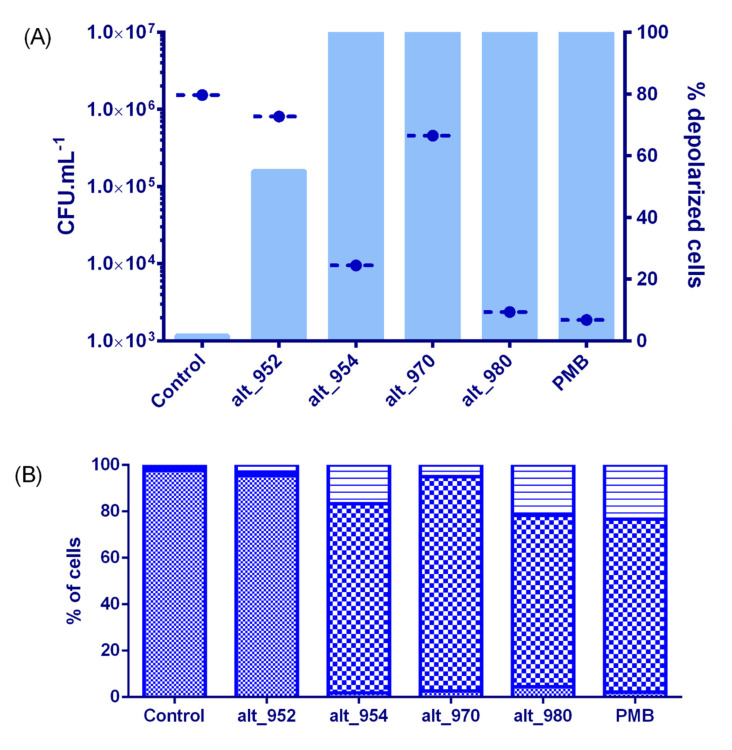
Physiological impact of alterins at 20 µM on *Vibrio tasmaniensis* LGP32 after a 4h-long incubation in sea salts (3%): (**A**) viable cells expressed as CFU.mL^−1^ (●) and cells exhibiting a depolarized membrane (histograms (■)), (**B**) intact cells (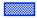) and cells exhibiting an altered membrane with low (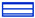) and high (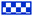) nucleic acid content.

As a first step, we have measured the antimicrobial activity of alt_952, alt_954, alt_970 and alt_980 against *V. tasmaniensis* LGP32 in seawater after a 4 hour-long incubation. It was performed at 20 µM, a concentration higher than MICs of alt_954 (2.7 µM) alt_980 (2.9 µM) and alt_970 (8.8 µM) and lower than the MIC of alt_952 (50 µM). The effects on membrane were also investigated under the same experimental conditions using live/dead bacterial viability kit for flow cytometry (Figure 6). A net cultivability loss of *V. tasmaniensis* (2-log reduction) was observed when incubated with alt_954 and alt_980 indicative of a bactericidal effect (Figure 7A). In these conditions, *V. tasmaniensis* was much less affected by alt_970 (1-log reduction) and not by alt_952 (Figure 7A). 

To assess alterin physiological impact on Gram-negative cells, flow cytometry analyses were implemented using Live/Dead cell viability assay and the membrane potential dye, DiBAC_4_(3) on *V. tasmaniensis* LGP32 treated as previously (i.e., 4 h in sterile seawater at 20 µM of alterins). Overall, such treatments resulted in a total loss of membrane integrity (Figure 6) for all tested alterins except alt_952. Nevertheless, even if no antibacterial effect is shown at 20µM for alt_952, around 55% of the treated cells present a depolarized membrane. Altogether, these results emphasize the membrane-directed mechanism of action of alterins. Moreover, two subpopulations of membrane-altered *Vibrio* were pointed out exhibiting low- and high-nucleic acid content populations [26] (Figure 6), suggesting different bacterial metabolic activity [27].

Experiments were then conducted using 300 µM of alt_952 that is to say at the same MIC factor as the one used for alt_954 and alt_980. The cultivability loss of *V. tasmaniensis* was identical to those observed using alt_954 and alt_980 at 20 µM (not shown). Flow cytometry analyses also revealed membrane perturbation and 100% of cells depolarization, emphasizing the alterins dose-dependent effects (Figure 7). 

Therefore, we conclude that the alterins provoke the membrane depolarization and lead to the bacteria death. Furthermore, the quantitative comparison of the alterins effects onto *V. tasmaniensis* LGP32 is in good agreement with the structure/activity relationship deduced from the MICs values and consistent with the LPS binding assays.

## 3. Discussion

We previously investigated the culturable microbiota in hemolymph of healthy molluscs and focused on its antimicrobial properties [12,13]. Whatever the marine animal explored, the antimicrobial culturable microbiota constituted between 3 to 20% of the strains assayed depending on the animal collected [12,13,28]. These antimicrobial compound producing strains were largely dominated by γ-Proteobacteria mainly affiliated to the genera *Vibrio* and *Pseudoalteromonas*. 

*Pseudoalteromonas* strains *hCg*-6 and *hCg*-42, which inhabit the hemolymph of healthy oysters [12] were shown to produce a family of cationic cyclolipopeptides with antibacterial activity. These CLPs are made of a heptapeptidic cycle connected to a hydrocarbon tail through an amido link. The peptidic cycle exhibits a net positive charge with a very polar sector made of the cationic side chains of two OH-Dab and one Arg while the fourth cationic charge (Dab) is surrounded by apolar residues (Leu, Dhb) and the hydrocarbon tail. The hydrocarbon tail varies in length (from 8 to 12 C), unsaturation and hydroxylation leading to a family of at least seven CLPs ranging from 926 to 982 Da. The *β*-OH-Dab residue is quite unusual even in non-ribosomal peptides. We examined the Norine Database [29] for such a residue without success. However, this residue has already been described in odilorhabdins [19] and ogipeptins [20]. The formers are produced by a nematode-symbiotic bacterium *Xenorhabdus nematophila* and are linear undecapeptides containing 2 *β*-OH-Dab residues at positions 2 and 3. Their antibiotic activity results from protein synthesis inhibition in target bacteria [19]. The ogipeptins are produced by *Pseudoalteromonas* SANK71903, a phylogenetically distant strain from the strains *hCg*-6 and *hCg*-42 (Figure 1). Ogipeptins, firstly denominated B-5529 substances in 2005 (Japan patent JP2005298434A) and renamed in 2016, are structurally related to CLPs. They were named ogipeptins referring to the location of the strain isolation, at Ogi-machi on the Sado island (Japan) [20]. The ogipeptins exhibit the same cationic heptapetidic ring except for the Dhb residue which exhibited a *trans* stereochemistry in its side chain. The strains SANK71903, *hCg*-6 and *hCg*-42 produce a different repertoire of CLPs. Some are common to all strains while other are strain specific (Table 1).

Since the cyclolipopeptide-producing strains have been isolated from bivalve hemolymph collected in the Atlantic ocean (Brittany, France [12]) and from algae in the Sea of Japan [20], producing-strains of *Pseudoalteromonas* appear to be widely distributed around the seas. Therefore, we propose herein to rename this family of CLPs, alterins, by reference to the common producing genus, *Pseudoalteromonas* and without taking their geographic origin into account. 

At the 3D structural level, the alterins are related to PMB behaviour. Indeed, in a similar way, no stable structure is observed in water, whereas a structure is obtained (using analysis of transferred NOE cross-peaks) in presence of LPS. Such an approach has been performed several times for PMB [30,31,32,33]. The amphipathic character of the alterin was less pronounced than for PMB with mainly the fatty acid side chain and the leucine residue. At the functional level, alterins and PMB exhibited similar modes of action and overall a quite similar antibiotic potency, except against some specific *Vibrio* species and *Pseudomonas aeruginosa* (see below). Indeed, firstly alterins unambiguously bind to LPS and then probably interact with the plasmic membrane resulting in its permeabilization and leading to bacterial lysis. This mechanism of action appears similar to that of PMB [34]. 

However, the alterins have not met all the structural key domains identified in structure activity studies of PMB known as (i) the distribution of the cationic charges, (ii) the linear tripeptide segment which is essential for polymyxin activity [35] and absent in alterin. It may explain the significant MIC difference between alterins and PMB in few cases such as against *Vibrio parahaemolyticus*13-028A/3.

Moreover, although the *Pseudoalteromonas* are known to be sensitive to PMB [36], the alterin-producing strains *hCg*-6 and *hCg*-42 were interestingly polymyxin-resistant. Since polymyxin is a well-known LPS-binding peptide produced by a Gram-positive bacterium [37], this may be indicative of different LPS structure/modification in the outer membrane of the producing-strains. Moreover, the prolific production of alterins by the strains *hCg*-6 and *hCg*-42 may extent the spectrum of antibacterial activity of these strains.

The alterins isolated from *Pseudoalteromonas* SANK 71903 were previously identified as drug candidates that inhibit the immunostimulatory functions of LPS. They have been shown to inhibit the LPS binding to CD14 and hence they were described as LPS inhibitors [38]. Using the same methodology, the pedopeptins were isolated from the strains *Pedobacter* SANK 72003. The pedopeptins include three similar cyclic depsipeptides (MW ranging from 1098.6 to 1114.6 Da) made of a nonapeptidic macrocycle alternating charged and apolar amino acid residues [39,40]. The pedopeptins exhibited a 10 fold-higher inhibition of LPS-induced TNF-α production than the alterin one [38,40]. By using the LPS of the same origin (*E. coli* O111:B4), we established the LPS-binding properties of alterins using both functional (LAL assay) and physicochemical (SPR) assays. Such a direct interaction may result from both electrostatic and hydrophobic bonding as described previously for PMB despite the structural divergences [41]. Therefore, the CD14 recognition of LPS which involved a hydrophobic pocket and a cationic segment may be unable to interact with the LPS-alterin complex as described for PMB [42]. This stresses that the CLPs from *Pseudoalteromonas* must be considered as antimicrobial LPS-binding peptides instead of LPS inhibitors. LPS-binding is a well-documented strategy in the antimicrobial arsenal. Indeed, almost all classes of antimicrobial peptides have been described as LPS-binders including the well-known NRPs polymyxins from the bacteria, *Paenibacillus polymyxa* [43], the anti-LPS-factors (ALF) from crustaceans [44], the small α-helical temporins from frogs [45] and the human cathelicidins [46]. However, this study exemplifies a LPS-binding strategy setting up by Gram-negative bacteria in microbial warfare.

Interestingly, these marine CLPs were much more potent than PMB against two marine pathogenic bacteria, *Vibrio harveyi* ORM4 and *Vibrio parahaemolyticus* 13-028A/3, respectively involved in mass mortality of the abalones, *Haliotis tuberculata* and the shrimps, *Litopenaeus vannamei* and *Penaeus monodon* [47,48]. Do CLPs evolve to tackle marine pathogenic bacteria and, if so, do they help to produce strains to colonize specific marine ecological niches such as bivalves? Further experiments are required to better understand how CLPs are or not key players in the non-host derived immunity. Due to the mechanism of action described above, we suspect the LPS structure to govern the susceptibility of target bacteria to alterins. The LPS structural characterization from both the producing-*Pseudoalteromonas* (insensitive to their produced CLPs) and susceptible strains will provide new insights into alterin/LPS interactions and therefore increased our understanding of their role in host-microbiota communication. Moreover, *Pseudoalteromonas* strains are suspected for a long time to play a significant ecological role [14]. The producing-strains investigated in this study were hosted by healthy bivalves. The study of the CLPs prevalence in host-associated *Pseudoalteromonas* may help to evaluate the impact of such compounds on host-microbiota shaping. How these strains are tolerated is a relevant question. A particular LPS structure may allow to evade the pattern recognition receptors and constitutive antimicrobial peptides [49]. In that context, CLPs and/or h*Cg*-6 and h*Cg*-42 may be used as a biomarker and molecular tools to investigate the molecular cross-talk between the bacteria themselves into the microbiota and between bacteria and the host.

Finally, this study exemplifies host-associated bacteria as an under-explored ecosystem deposit of new bioactive metabolites to renew the antimicrobial arsenal. In the alarming landscape of the AntiMicrobial Resistance crisis, symbiont and/or associated microbiota may be a useful biological starting material to improve the weakly flowing antibiotics pipeline. Indeed, beyond the anti-inflammatory activities [38], the alterins may act as potent and/or synergistic antibiotic peptides or even as LPS neutralizing peptides. 

## 4. Materials and Methods

### 4.1. Bacterial Strains and Growth Conditions

The bacterial strains *Aeromonas caviae* CIP 7616, *Aeromonas hydrophila* CIP 7614, *Escherichia coli* ML35, *Escherichia coli* SBS 363, *Salmonella enterica* CIP 8297 and *Yersinia ruckeri* ATCC 29473 were grown in Tryptic Soy Broth. The marine bacterial strains *Vibrio crassostreae*J2–9, *Vibrio tasmaniensis*LGP32, *Vibrio tapetis* CECT 4600 were grown in Marine Broth. All of them were cultivated at optimal temperature according to ATCC and CIP specifications. The strain *Vibrio parahaemolyticus*13-028A/3 was grown at 28 °C in Tryptic Soy Broth supplemented in NaCl (20 g.L^−1^) according to [47]. The strains *Pseudoalteromonas* sp. *hCg*-6 and *hCg*-42 were cultured using a synthetic medium containing: 17.11 g sucrose, 30 g sea salts (Sigma-S9883), 7.6 g K_2_HPO_4_, 3 g KH_2_PO_4_ and 20 mL MEM 50X (Dulbecco) per liter. The cultures were grown at 18 °C for 72 h with shaking (100 rpm).

### 4.2. Antibacterial Compounds Isolation

The cell-free supernatants of *Pseudoalteromonas hCg*-6 and *hCg*-42 cultures were collected after centrifugation (6000× *g* for 30 min at 4 °C). Antibacterial compounds were purified using a two-step protocol. Cell-free supernatants were directly subjected and fractionated onto solid-phase extraction (SPE) C18 column (UptiClean C18-S, Interchim, Montluçon, France) equilibrated with 10% Acetonitrile (ACN), 0.07% trifluoro-acetic acid (TFA). Elution was performed sequentially with 10% and 40% ACN, 0.07%TFA. The latter SPE fraction was analyzed using UPLC-MS (Acquity H-Class Bio (Waters) combined with a Quattromicro (Micromass)). For purification, the SPE fraction was loaded onto semi-preparative C18-column (HTec, 250 × 10 mm, Macherey-Nagel, Dueren, Germany). Elution was performed with a bi-linear gradient from 20 to 40% ACN 0.07% TFA over 45 min at 40 °C and monitored at 220 and 280 nm. Manually collected peaks were lyophilized, reconstituted in ultra-pure water (Merck MilliQ, Millipore, Burlington, Massachusetts, USA) and then assayed for antibacterial activity.

### 4.3. NMR Experiments

A 500 MHz Bruker spectrometer equipped with a cryo-probe and pulsed-field gradients was used to record all NMR spectra. Spectra acquisition and data processing was performed on a Linux workstation with the TopSpin software (Bruker Biospin). The internal reference used for the calibration of proton chemical shifts was TSP (TrimethylSilyl Propionate). All spectra were recorded at concentration around 5 mM dissolved in aqueous (90% H_2_O, 10% D_2_O), the pH is adjusted to 4.5. Homonuclear 2-D spectra DQF-COSY, TOCSY and ROESY were recorded at 298 K in the phase-sensitive mode using the States-TPPI method as data matrices of 512 real (t1) × 4K (t2) complex data points; from 16 to 48 scans per t1 increment with 1.2 s recovery delay and spectral width of 5341 Hz in both dimensions were used. For acquisition of the two-dimensional transferred NOESY spectrum, a solution of LPS was added on the aqueous solution of the cyclolipopeptide CLP980, to a final concentration of 75 µM corresponding to a peptide: LPS molar ratio of 70:1. The parameters for the Tr-NOESY spectrum are unchanged except for the mixing time which was 100 ms.

### 4.4. Structure Calculations

The three-dimensional structures of the CLP were calculated with the AMBER software [50]. From the two-dimensional spectrum, Tr-NOESY NOE cross-peaks were integrated within the NMRView software [51]. The volumes of NOE peaks between methylene pair protons were used as a reference of 1.8 Å. Then based on the intensities of the NOEs, the distance constraints were evaluated. For distance estimation, NOEs were raised to upper limits, 2.7 Å for strong NOEs, 3.3 Å for medium NOEs and 5 Å for low NOEs. A total of 100 structures were calculated by AMBER software following the previously reported procedure [52]. Briefly, the initial cooking stage was performed at 1000 K to generate 100 initial random structures. Simulated annealing calculations were then done during 20 ps (20,000 steps of 1 fs). The temperature was raised to 1000 K and kept constant for the first 5000 steps. Then the system was gradually cooled down to 100 K from step 5001 to 18,000. The temperature was then brought to 0 K during the remaining 2000 steps. The force constant of the distance restraints was set to 2.0 kcal mol^−1^.Å during the first 3000 steps, and then increased gradually to 20 kcal mol^−1^ Å. for the remaining steps 3001 to 20,000. The 20 lowest energy structures with no violations >0.3 Å were representative of the compound structure. The representation and quantitative analysis were done using either MOLMOL [53] or YASARA [54]. The ptraj program was used to measure the dihedral angles.

### 4.5. Minimal Inhibitory Concentration (MIC)

MICs were determined using broth microdilution method according to [21]. To resume, antibacterial compounds ranging from 0.1 to 100 µM were assayed against the target bacteria in mid-logarithmic phase culture (1.10^6^ UFC.mL^−1^) in adapted culture medium according to the strain. MICs were examined by unaided eye after a 48h-incubation time at the optimal temperature growth. The lipopeptide PMB (Sigma-P4932) was used as antibiotic control. 

### 4.6. Effect of Alterins on Vibrio Tasmaniensis Viability

Sterile seawater containing alterin (20 µM) was inoculated with the target bacteria at 1.10^6^ UFC mL^−1^. After a 4 h-long incubation, viable bacteria were counted. Diluted samples (50 µL) in sterile seawater were spread in duplicate on appropriate medium. Cultivable bacterial concentration (CFU mL^−1^) was determined after overnight incubation at optimal growth temperature.

### 4.7. Flow Cytometry

Bacterial cells were enumerated by flow cytometry according to the protocol described by Marie et al [55], slightly modified by Bouvy et al [56] with the use of higher fluochrome concentration. Samples were incubated with SYBR Green I (Molecular Probes, Eugene, OR, USA) at a final concentration of 1/375 for 15 min at 4 °C in the dark. Counts were performed using a FACS Calibur flow cytometer (Becton Dickinson, San Jose, CA, USA) equipped with an air-cooled argon laser (488 nm, 15 mW). Stained bacterial cells, excited at 488 nm, were enumerated according to the side scatter (SSC) and green fluorescence (FL1) was measured using a 530/30 nm filter. These cell parameters were recorded on a four-decade logarithmic scale mapped onto 1024 channels and analysed with CellQuestPro software.

To investigate membrane integrity of the target *Vibrio tasmaniensis* LGP32, cells were stained using a Live/Dead BacLight bacterial-viability kit (Molecular Probes) according to the manufacturer’s recommendations, by adding 3 µL of a 1:1 (*v*/*v*) mixture of SYTO9 and PI to the bacterial suspension. Incubation was performed for 15 min in the dark, at room temperature. Samples were then analyzed with a FACS Calibur flow cytometer. Analyses were run at low speed (15 μL min^−1^) for a 2 min acquisition time. The green fluorescence of SYTO9 was measured at 530 nm (FL1 channel), and the red fluorescence of PI was measured above 670 nm (FL3 channel). Cells with damaged membrane (PI-positive cells) were differentiated from those with intact membrane by their signature in a plot of green versus red fluorescence.

The membrane potential of the target bacteria was analysed using the potential-sensitive probe Bis-1,3-Dibutylbarbituric AcideTrimethineOxonol, (DiBAC_4_(3), MolecularProbes). DiBAC_4_(3) was reconstituted in DMSO stock solution at 1mM and stocked at −20 °C until use. Samples (500 µL) were incubated with 1 µL of DiBAC_4_(3) stock solution for 10mn in the dark [57,58]. Under the effect of depolarization cells accumulate DiBAC_4_(3), fluoresce bright green and can easily be detected by flow cytometry. After excitation at 488 nm cells with depolarized membrane become fluorescent and reemit at 516 nm recovery of the signal at FL1. Positive control of the initial sample is carried out with 20% alcohol.

### 4.8. LPS Binding Assays

The CLPs were assayed for their LPS-binding properties by using the QCL-1000 *Limulus* Amoebocyte Lysate kit (LAL-Lonza, Bâle, Switzerland) according to manufacturer instructions. Briefly, commercial LPS at 1EU.mL^−1^ from *Escherichia coli* O111:B4 was previously incubated with CLPs ranging from 0.01 to 10 µM for 15 min at 37 °C. Free LPS was then quantified according to the manufacturer’s instructions. PMB was used as control. 

Surface plasmon resonance experiments were carried out at 25 °C using a BIACORE 3000 apparatus (GE Healthcare, Uppsala, Sweden). The CLPs were immobilized (500–600 RU) on different flowcell of a carboxylic dextran CM5 sensor chip (GE Healthcare, Uppsala, Sweden) *via* primary amino groups according to the manufacturer’s instructions. The running buffer was 10 mM Hepes (pH 7.4), 150 mM NaCl, and 3 mM EDTA. For binding experiments, LPS from *E. coli* O111:B4 (Sigma) was used. Lipids were sonicated (15 min, 25 °C) and injected simultaneously at different concentrations into the measuring and control (no protein immobilized) flow cells at a high flow rate (50 μL/min) to limit mass transport effect. Sensor surface was regenerated using a short pulse of 0.02% SDS. The binding level in RU was measured at the end of the binding after normalization to the same CLP coated level. 

## Figures and Tables

**Table 1:** Alterin hydrocarbon tail, molecular weight and producing-*Pseudoalteromonas* strains.

	Denomination	Hydrocarbon Tail	MW (Da)	*Pseudoalteromonas* Strains		
				***hCg*-6**	***hCg*-42**	**SANK 71903**
	alt_926	C_8:0_	926	+	+	−
	alt_940	C_9:0_	940	+	+	−
	alt_952	*cis* C_10:1_Δ^5^	952	+	+	−
	alt_954	C_10:0_	954	+	+	+
Alterin	alt_970	3′-OH C_10:0_	970	+	+	−
	alt_980	*cis* C_12:1_ Δ^5^	980	+	+	+
	alt_982	C_12:0_	982	+	−	+
	alt_1008	*cis* C_14:1_Δ^7^	1008	−	−	+

**Table 2:** Minimal inhibitory concentrations (µM) of alterins against a panel of Gram-negative target bacteria.

		MICs (µM)							
		Alterins							PMB.
MW (Da)		926	940	954	952	970	980	982	
Hydrocarbon Tail		C_8:0_	C_9:0_	C_10:0_	C_10:1_	3′OH-C_10:0_	C_12:1_	C_12:0_	
*A. caviae*	CIP 7616			5.5	ND	17.6	5.8	ND	3.1
*A. hydrophila*	CIP 7614	>100	25	2.7	ND	ND	2.9	ND	0.8
*E. coli*	ML35	100	50	1.4	25	35	1.5	ND	3.1
*E. coli*	SBS363	50	25	0.2	0.8	0.1	0.4	6.3	0.2
*P. aeruginosa*	ATCC 27853	>100	50	22	50	35.2	23	ND	1.6
*Pseudoalteromonas*	h*Cg*-6	>100	>100	>100	>100	>100	>100	ND	>100
*Pseudoalteromonas*	h*Cg*-42	ND	ND	>100	>100	>100	>100	ND	>100
*S. enterica*	CIP 8297	12.5	6.25	0.7	6.25	1.1	1.4	ND	0.8
*V. harveyi*	ORM4	50	6.25	1.4	12.5	ND	1.4	ND	25
*V. parahaemolyticus*	13-028A/3	ND	ND	6.25	ND	6.25	6.25	ND	>100
*V. tapetis*	CECT 4600^T^	ND	ND	>100	>100	>100	>100	ND	>100
*V. tasmaniensis*	LGP 32	50	6.25	2.7	50	8.8	2.9	ND	3
*Y. ruckeri*	ATCC 29473	50	6.25	0.3	0.4	1.1	0.4	12.5	0.4

ND Stands for *Not Determined*.

## Patents

This work has resulted in a patent “Use of a bacterium isolated from the genus *Pseudoalteromonas*, Cyclolipopeptides and uses thereof” (FR3005963B1 and PCT WO2014187993A1).
